# Comprehensive use of a high-frequency electric knife, balloon dilatation, and cryotherapy for tuberculous central tracheobronchial cicatricial constriction

**DOI:** 10.1186/s12893-022-01862-y

**Published:** 2022-12-02

**Authors:** Kunying Li, Taomei Lian, Qiang Liang, Liping Zhang, Ying Chen, Jiwei Liu, Jiaping Qian, Xin Liu

**Affiliations:** 1 Endoscopy Center, Henan Provincial Chest Hospital, Zhengzhou University, Zhengzhou, 450000 China; 2 Department of Tuberculosis, Henan Provincial Chest Hospital, Zhengzhou University, Zhengzhou, 450000 China; 3 Department of Infectious Diseases, Sanmenxia Central Hospital, Sanmenxia, Henan China; 4 Department of Medical Imaging, Henan Provincial Chest Hospital, Zhengzhou University, Zhengzhou, 450000 China

**Keywords:** Tracheobronchial stenosis, Dilatation, Cryotherapy, Electrocautery, Recurrence, Complications

## Abstract

**Background:**

To examine the benefits of interventional therapy for cicatricial constriction using a high-frequency electric knife, saccular dilatation, and cryotherapy.

**Methods:**

This case series included patients with central tracheobronchial cicatricial constriction admitted to the Department of Tuberculosis of Henan Provincial Chest Hospital from July 2018 to March 2021 and treated with bronchoscopic interventional therapies based on systemic anti-tuberculosis treatment.

**Results:**

96 patients were included, in whom 443 interventional therapies were performed. The total mid-(3 months) and long-term (12 months) effective rates were both 100%. The internal diameter of tracheobronchial stenosis increased after the operation, and the difference was statistically significant (all < 0.05). After interventional treatment, patients’ symptoms of choking sensation in the chest and shortness of breath were relieved. Respiratory function was obviously improved. The ratios of hemorrhage, granulation hyperplasia, chest pain, and postoperative fever were 58.2%, 42.6%, 31.3%, and 26.7%, respectively. No focal transmission and progression of tuberculosis occurred, and no serious complications were observed.

**Conclusion:**

The use of a high-frequency electric knife, saccular dilatation, and/or cryotherapy according to the pathological stage of the tracheobronchial cicatricial constriction is feasible, with good mid- and long-term curative effects and few complications.

## Background

Benign central tracheobronchial stenosis refers to tracheobronchial stenosis caused by various benign pathological changes in the trachea, left and right primary bronchi, and right middle part of the bronchus due to stenosis after endotracheal intubation, tracheostomy tube placement, surgery, infections, inflammatory disorders, extrinsic compression, benign endobronchial tumors, and tracheobronchomalacia [1–5]. It is a debilitating condition that can seriously affect the patients’ quality of life and can lead to different degrees of expiratory dyspnea or even death by suffocation [1, 2]. Tracheobronchial stenosis with cicatricial contracture is the most common acquired benign tracheobronchial stenosis, mainly caused by cicatricial constriction. Unfortunately, medical treatments are mostly ineffective, and the condition is prone to recurrences [1–5]. It is clearly pointed out in the guideline for the diagnosis and treatment of tracheobronchial tuberculosis (Trial) that anti-tuberculosis treatment combined with tracheoscopic airway interventional therapy can improve the therapeutic effect of tracheobronchial tuberculosis and reduce the various complications and sequelae caused by it, and, more importantly, it can help preserve the lung function of the patients to the greatest extent and effectively solve some problems that traditional anti-tuberculosis drugs cannot solve.

In the past, tracheobronchial stenosis was mostly treated by surgical resection and end-to-end anastomosis or reconstruction of the trachea [6–8]. In recent years, with the development of interventional pneumology and technologies such as balloon dilatation, high-frequency electric knife, laser, argon plasma coagulation (APC), cryotherapy, and tracheobronchial stent implantation [9, 10], the treatment paradigm shifted for benign tracheobronchial stenosis from surgery to bronchoscopic interventional therapy, except that surgical treatment cannot be avoided in the end stages of bronchostenosis [11–13]. Selecting the appropriate treatment for benign tracheobronchial stenosis is of great importance [12].

Mechanical dilatation is a means to open strictures, but it should not be used alone because of the risk of additional cicatricial tissue formation [9, 10]. Electrocautery can be used to remove excess cicatricial tissues, but it induces trauma and tissue damage and, again, can lead to additional tissue formation [9, 10]. Cryotherapy was proposed in the late twentieth century to manage carcinoma-induced laryngotracheobronchial strictures [14, 15]. Advances in the technique allow the delivery of liquid nitrogen through a catheter to cause tissue ablation through flash freezing [16]. The initial use of cryotherapy for airway strictures reported significant complications due to nitrogen gas expansion [17–20], but recent systems improved the procedure’s safety [21]. Therefore, multimodal management of tracheobronchial stricture might be the key to avoiding recurrences. Cryotherapy is thought to be effective in tissues with relatively high water content. Its principle is the use of cryogenic materials and equipment to produce ultra-low temperatures so that the water molecules in the tissue cells quickly crystallize into ice, breaking the cells apart, stopping the local blood flow, and causing microthrombosis and other chronic pathological processes, ultimately leading to necrosis. The effect of cryotherapy is weak, and the local reaction is light, which is easily accepted by patients. Cryotherapy generally does not damage airway cartilage and rarely causes airway perforation. After treatment, granulation tissue hyperplasia and fibrous scar formation rate are low.

Therefore, this case series study aimed to examine the benefits of interventional therapy for cicatricial constriction using a high-frequency electric knife, saccular dilatation, and cryotherapy.

## Methods

### Study design and patients

This case series included patients with central tracheobronchial cicatricial constriction admitted to the Department of Tuberculosis of Henan Provincial Chest Hospital from July 2018 to March 2021 and treated with bronchoscopic interventional therapies based on systemic anti-tuberculosis treatment. All patients signed the informed consent form and underwent bronchoscopy.

The inclusion criteria were (1) 18–60 years of age, (2) diagnosed with tuberculosis according to positive sputum or sputum culture [22], with results of bronchoscopy showed bronchial tuberculosis with cicatricial constriction and stenosis ≥ 50%, (3) atelectasis for < 3 months and preserved lung functions, (4) positive sputum culture for *Mycobacterium tuberculosis*, (5) underwent bronchoscopic interventional therapy, (6) follow-up of ≥ 12 months, and (7) all patients received medical anti-tuberculosis treatment.

The exclusion criteria were (1) intracavitary stenosis, dynamic stenosis, stenosis caused by external pressure, or stenosis caused by tracheobronchial softening, (2) irregular anti-tuberculosis treatment or stopped anti-tuberculosis medication for > 1 month, or (3) tuberculosis resistant to multiple drugs.

### Typical treatment procedure

Before and after bronchoscopic interventional treatment, all patients underwent routine lung high-resolution computed tomography (CT) (HRCT) (Philips, Best, The Netherlands): tube voltage of 120 kV, tube current of 600 mA, thickness of 1 mm, and scanning time 500 ms. The patients were lying flat on the table for HRCT, and a three-dimensional reconstruction of the trachea was made on the computer after HRCT examination of the lung. And all patients underwent routine Pulmonary function tests, Forced expiratory volume in 1 s (FEV1) and peak flow velocity (PEF) were measured with CUST0 vitm pulmonary function meter. Bronchoscopy (BF260 electronic bronchoscope, Olympus Corporation, Tokyo, Japan) was performed to evaluate the length and degree of the central tracheobronchial stenosis. According to the different stages of pathological changes of the cicatricial constriction and the actual manifestations of patients through bronchoscopy, the patients were roughly grouped and managed as follows. In the stage of scars hyperplasia, when the mucous membranes of the tracheobronchial walls were congestive, dropsical, and asperous, with caseous necrosis almost disappearing, cryotherapy was mainly performed, with saccular dilatation if necessary. In the period of repair under scar formation, when the caseous necrosis on the mucous membranes of the tracheobronchial walls completely disappeared, the mucous membranes of the tube walls were not as dropsical as before, and the superficial scars were changed, with no more acute inflammations; saccular dilatation and sequential cryotherapy were performed. In the mature stage of the scars, when the tube wall was thickened, the scars on the mucous membrane were obvious, which were white and not congestive, with no caseous necrosis and hyperplasia of granulation; then, high-frequency electric knife, saccular dilatation, and sequential cryotherapy were used. The first 32 patients in each phase treated during the study period were included. All patients were treated with interventional therapies by the full-time medical team in the bronchoscopy room.

An ERBE cryotherapy equipment and soft flexible cryoprobe with a diameter of 1.9 mm and a probe measuring 10 mm were used (Erbe Elektromedizin GmbH, Tuebingen, Germany), with liquid CO_2_ as the freezing source. The multi-point freezing method was adopted, 30–120 s for each part. Saccular dilatation was performed using a three-level tracheobronchial Foley’s tube with outer diameters of 8, 9, and 10 mm (Nanjing Micro-Tech Endoscopy Group, Nanjing, China). A pressure pump was used to press the saccule from low to high. The pressure was 4–10 atm for 60–180 s, and each part was dilated one or two times. A high-frequency electric knife (SurgMaster UES-40 high-frequency electric knife, Olympus Corporation, Tokyo, Japan) and supporting acusector were used to relieve the scars, from shallow to deep, about 5 mm deep every time, and saccular dilatation was performed afterward. The frequency of examination and treatment of all patients was gradually prolonged, from once a week to once every 2 weeks to once every 3 weeks. According to the changes in patients’ conditions and their pathological stage, the treatment time and plan were properly adjusted. After their conditions were stable, the patients were re-examined after 3 months and 1 year to judge the mid- and long-term curative effects.

The inner diameter of tracheobronchial stenosis was measured by the scale plate of the bronchoscope with the chest CT image: the inner diameter of tracheobronchial stenosis was calculated as (horizontal diameter + longitudinal diameter)/2. When the bronchoscope (diameter of 4.9 mm) could not advance in the bronchial stenosis, the length of the bronchoscope entering the body was recorded from the nose or mouth, as appropriate. The tracheobronchial stenosis, distal lung conditions, the diseased bronchial wall with or without calcification, and the surrounding blood vessels were observed. The relevant data was measured, mainly the length of the tracheobronchial stenosis and the diameter of the narrowest part.

The diameter of tracheobronchial stenosis of all patients before and after the first interventional therapy and at the mid- and long-term follow-ups was recorded. The numbers of interventional therapies, including cryotherapy, balloon dilatation, and high-frequency electric knife needed to achieve significant therapeutic effect were recorded, and the incidence of complications such as bleeding, mediastinal emphysema, and tracheobronchial perforation, thoracalgia, and granulation hyperplasia after interventional therapies. The recurrence of tracheobronchial stenosis at 3 and 12 months was recorded.

Completely effective therapy was defined as a recovery of the internal diameter of the constrictive trachea to > 80%. Significantly effective was defined as the internal diameter of the constrictive trachea recovered to 50–79%. Slightly effective was defined as stenosis still of > 50%, but the bronchoscope (with a diameter of 4.9 mm) could pass smoothly. Ineffective was defined as no improvement, and the bronchoscope could not pass [19, 21]. Total effective rate (%) = (completely effective cases + significantly effective cases + slightly effective cases)/total cases × 100%. Recurrence of tracheobronchial restenosis after interventional therapy was defined as the diameter of the constrictive trachea returning to the state before dilatation or was even narrower than before [19, 21].

### Statistical analysis

SPSS 19.0 (IBM Corp., Armonk, NY, USA) was used for statistical analysis. Continuous data were expressed as means ± standard deviation. Repeated measurement analysis of variance was used to compare the tracheobronchial diameter before treatment and after the operation. Bonferroni’s method to correct the P-value for the post hoc analyses. The categorical data were presented as n (%) and analyzed using the Chi-square test. P-values < 0.05 were considered statistically significant.

## Results

### Characteristics of the patients

This study included 96 patients aged from 22 to 54 years old, with an average age of 28.3 ± 8.1 years, and including 42 males and 54 females. All patients had tracheobronchial tuberculosis. The stenosis sites were the left primary bronchus for 47, the right primary bronchus for 31, and the right middle bronchus for 18 (Table 1).

**Table 1:** Characteristics of the patients

Variables	Values
Sex, n (%)	
Male	42 (43.8)
Female	54 (56.2)
Age (years, mean ± SD)	28.3 ± 8.1
Site of tuberculosis stenosis, n (%)	
Left primary bronchus	47 (48.9)
Right primary bronchus	31 (32.3)
Right middle bronchus	18 (18.8)

### Curative effect

**Fig. 1:**
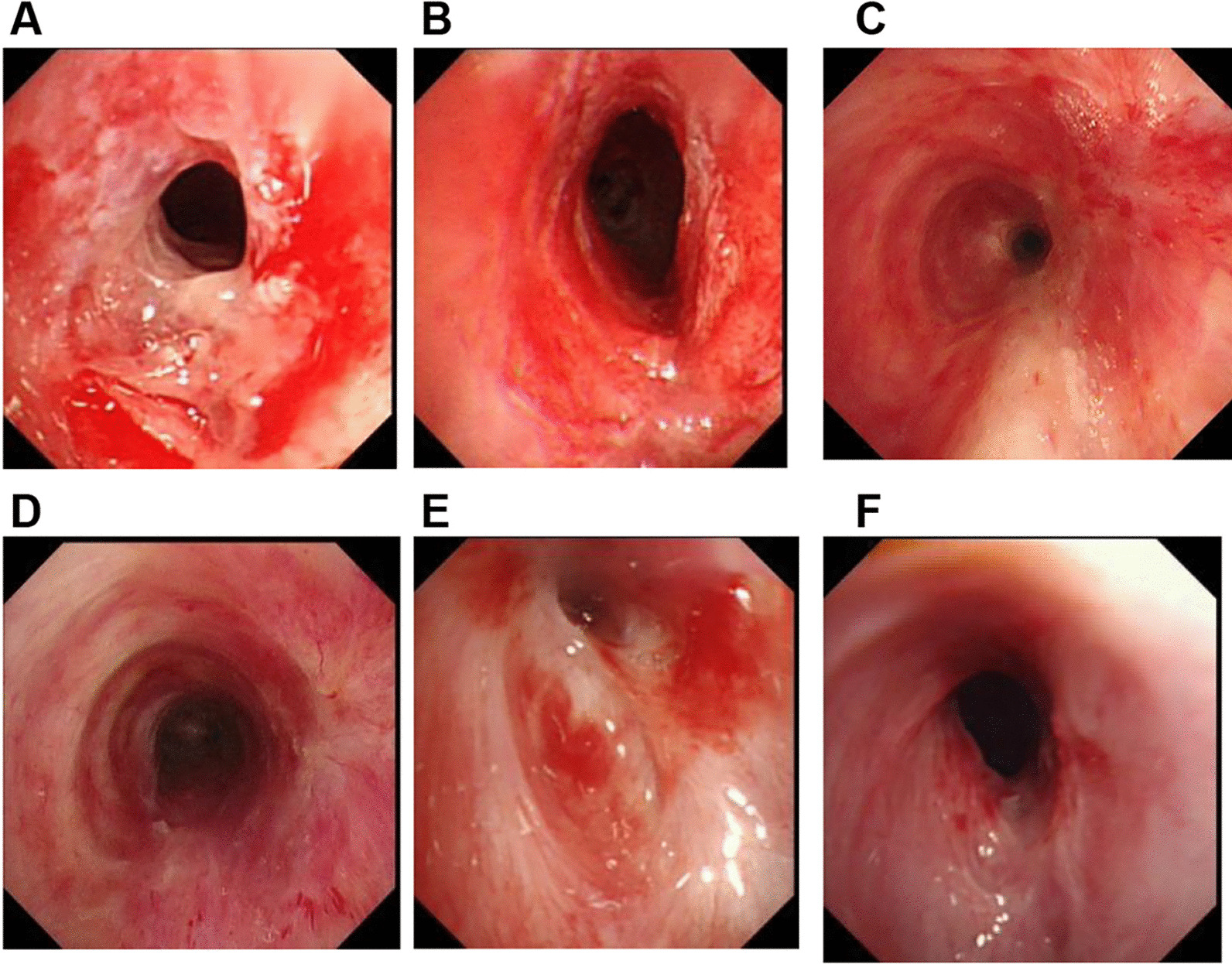
The images are representative pictures of each group: scar hyperplasia, repair under scar formation, and mature scar. **A** Before the treatment of scar hyperplasia. **B** After treatment of scar hyperplasia. **C** Before the treatment of repair under scar formation. **D** After treatment of repair under scar formation. **E** Before the treatment of mature scar. **F** After treatment of mature scar

A total of 443 interventional treatments were performed on the 96 patients, as shown in Table 2. A total of 143, 122, and 178 treatments were performed in the scar hyperplasia, repair under scar formation, and mature scar groups, respectively. The high-frequency electric knife had to be used 18 times. The curative effect during the follow-up period is shown in Table 3. The mid- and long-term effective rates were both 100%. The internal diameter of tracheobronchial stenosis increased after the operation (Table 4 and Fig. 1), Pulmonary function FEV1 and PEF were significantly improved (Tables 5 and 6) and the differences reach statistical significance (all P < 0.05) (Tables 7, 8 and 9). After interventional treatment, patients’ symptoms of choking sensation in the chest and shortness of breath were relieved to different degrees.

**Table 2:** Numbers of interventional treatments received by the patients with different stages of pathological changes of the scars

Group	Patients	Saccular dilatation (n)	Cryotherapy (n)	High-frequency electric knife (n)	Total interventional treatments (n)
Scar hyperplasia	32	19	124	0	143
Repair under scar formation	32	39	83	0	122
Mature scars	32	68	92	18	178

**Table 3:** Curative effect

Follow-up	Cases (n)	Completely effective (n)	Significantly effective (n)	Slightly effective (n)	Ineffective (n)	Total effective rate (%)
Mid-term	96	24	47	25	0	100
Long-term	96	21	49	26	0	100

Total effective rate (%) = (completely effective cases + significantly effective cases + slightly effective cases)/total cases × 100%

**Table 4:** Internal diameter of tracheal stenosis before and after treatment

Group	n	Internal diameter of tracheal stenosis before the first interventional therapy (mm, mean ± standard deviation)	Internal diameter of tracheal stenosis Mid-term (3 months after the operation) (mm, mean ± standard deviation)	Internal diameter of tracheal stenosis Long-term (12 months and above after the operation) (mm, mean ± standard deviation)
Scar hyperplasia	32	3.6 ± 0.6	6.6 ± 0.3	6.5 ± 0.4
Repair under scar formation	32	3.7 ± 0.5	6.8 ± 0.9	6.7 ± 0.7
Mature scars	32	3.6 ± 0.9	6.9 ± 0.7	6.7 ± 0.8

**Table 5:** Different stages of cicatricial lesions forced expiratory volume in 1 s (FEV1)

Group	n	Internal diameter of tracheal stenosis before the first interventional therapy (FEV1, L, mean ± standard deviation)	Internal diameter of tracheal stenosis Mid-term (3 months after the operation) (FEV1, L, mean ± standard deviation)	Internal diameter of tracheal stenosis Long-term (12 months and above after the operation) (FEV1,L, mean ± standard deviation)
Scar hyperplasia	32	1.37 ± 0.26	1.83 ± 0.32	1.82 ± 0.24
Repair under scar formation	32	1.45 ± 0.34	1.94 ± 0.21	1.91 ± 0.23
Mature scars	32	1.34 ± 0.41	1.87 ± 0.39	1.81 ± 0.44

**Table 6:** Different stages of cicatricial lesions peak expiratory flow

Group	n	Internal diameter of tracheal stenosis before the first interventional therapy (PEF, L/s, mean ± standard deviation)	Internal diameter of tracheal stenosis Mid-term (3 months after the operation) (PEF, L/s, mean ± standard deviation)	Internal diameter of tracheal stenosis Long-term (12 months and above after the operation) (PEF, L/s, mean ± standard deviation)
Scar hyperplasia	32	3.63 ± 0.37	5.14 ± 0.26	5.11 ± 0.18
Repair under scar formation	32	3.72 ± 0.31	5.16 ± 0.17	5.12 ± 0.21
Mature scars	32	3.61 ± 0.44	5.12 ± 0.31	5.08 ± 0.42

**Table 7:** Comparison of the internal diameter of tracheal stenosis before and after treatment

Variable	Wilks’ Lambda	F	P
Internal diameter of tracheal stenosis			
Scar hyperplasia (before treatment vs. Mid-term after treatment vs. Long-term after treatment)	0.037	3.913	0.000
Repair under scar formation(before treatment vs. Mid-term after treatment vs. Long-term after treatment)	0.029	6.079	0.000
Mature scars (before treatment vs. Mid-term after treatment vs. Long-term after treatment)	0.026	3.742	0.000

**Table 8:** Comparison of pulmonary function before and after treatment (FEV1)

Variable	Wilks’ Lambda	F	P
Internal diameter of tracheal stenosis			
Scar hyperplasia (before treatment vs. Mid-term after treatment vs. Long-term after treatment)	0.041	4.426	0.000
Repair under scar formation(before treatment vs. Mid-term after treatment vs. Long-term after treatment)	0.032	6.129	0.000
Mature scars (before treatment vs. Mid-term after treatment vs. Long-term after treatment)	0.054	3.862	0.000

**Table 9:** Comparison of the pulmonary function before and after treatment (PEF)

Variable	Wilks’ Lambda	F	P
Internal diameter of tracheal stenosis			
Scar hyperplasia (before treatment vs. Mid-term after treatment vs. Long-term after treatment)	0.033	3.533	0.000
Repair under scar formation (before treatment vs. Mid-term after treatment vs. Long-term after treatment)	0.027	6.464	0.000
Mature scars (before treatment vs. Mid-term after treatment vs. Long-term after treatment)	0.046	3.847	0.000

### Complications

The incidence of hemorrhage, granulation hyperplasia, chest pain, and postoperative fever was 58.2%, 42.6%, 31.3%, and 26.7%, respectively. No focal transmission and progression of tuberculosis occurred. After saccular dilatation, there were often different degrees of bleeding. Spraying a proper amount of cold saline water (4 °C) was sufficient to control active bleeding. There were no cases of severe hemoptysis, pneumothorax, and mediastinal emphysema. Some patients had throat and nose discomfort after the operation, most of which was relieved by themselves or relieved by inhaling budesonide after the operation. Twelve patients had a slight fever after the operation, and all of them recovered to normal within 3 days.

## Discussion

In the present study, all patients were followed for at least 12 months and achieved good clinical treatment results. The curative effect evaluation showed that after the tracheobronchial stenosis was relieved, the scar was formed and relatively stable, with a smooth surface. The mid- and long-term effects were both good. The results suggest that high-frequency electric knife, saccular dilatation, and/or cryotherapy according to the pathological stage of the tracheobronchial cicatricial constriction is feasible, with good mid- and long-term curative effects and few complications.

All treatment techniques have their advantages and disadvantages. The proper selection of interventional treatment techniques such as saccular dilatation, high-frequency electric knife, argon plasma coagulation (APC), cryotherapy, and tracheobronchial stent implantation according to the condition of the patient is of great importance to the treatment of benign tracheobronchial stenosis and lowering the incidence and degree of tracheobronchial restenosis. Because damage to the mucous membranes of the tracheobronchial walls will lead to subsequent local tissue hyperplasia, recovery and scar formation during interventional therapy can lead to restenosis in the mid and long term, with the need for repeated interventional therapy to relieve tracheobronchial stenosis, but still with a risk of restenosis. The proper selection of the management strategy is necessary to break the vicious cycle of “injury–recovery–stenosis–reinjury”. Cryotherapy might be ideal for breaking this cycle since tissue destruction by cryotherapy preserves the extracellular matrix and the regenerative properties of the tissues, which might allow healing with minimal fibrosis [16, 23]. In addition, cryotherapy might make the tissue more malleable and more atraumatic dilatation [24, 25]. A study that used cryotherapy followed by balloon dilatation showed a marked improvement in stenosis, with 85% of patients having grade 3–4 stenosis before treatment and 15% after treatment [13]. Cryotherapy uses the hypothermia effect to cause ischemic necrosis of the tissues. It is more sensitive to tissue with abundant water content and is less likely to cause granulation after treatment [1, 7]. The treatment rate of eight patients with granulation tissue hyperplasia was 100% without complication [26].

Previous studies reported severe complications in 6–19% of the cases [18, 24]. In the present study, only minor complications were observed, and all complications could be managed. Of note, the electric knife was used in only four treatments, which might decrease the risk of perforation. For scar contracture and strong scar toughness, the needle-shaped electric knife can be first used to cut to release the scar, and then balloon dilation treatment can be performed.

This study has limitations. The sample size was small, and all patients were from the same hospital, probably introducing bias. In addition, processes that could be involved in recurrence, such as systemic inflammation, were not examined. Clinical trials are necessary to determine the efficacy and safety of the methods proposed here.

## Conclusion

In conclusion, using a high-frequency electric knife balloon dilatation and/or cryotherapy to manage cicatricial tracheobronchial stenosis according to the different pathological stages of repair under scar formation might be beneficial by relieving tracheobronchial stenosis and reducing the additional damages and injuries brought by the interventional therapy.

## Data Availability

All of our data are included in the text.
